# Massively parallel whole genome amplification for single-cell sequencing using droplet microfluidics

**DOI:** 10.1038/s41598-017-05436-4

**Published:** 2017-07-12

**Authors:** Masahito Hosokawa, Yohei Nishikawa, Masato Kogawa, Haruko Takeyama

**Affiliations:** 1 Research Organization for Nano & Life Innovation, Waseda University, 513 Wasedatsurumaki-cho, Shinjuku-ku Tokyo, 162–0041 Japan; 2 PRESTO, Japan Science and Technology Agency (JST), 5-3 Yonban-cho, Chiyoda-ku Tokyo, 102–0075 Japan; 3 Department of Life Science and Medical Bioscience, Waseda University, 2-2 Wakamatsu-cho, Shinjuku-ku Tokyo, 162–8480 Japan; 4 Computational Bio Big-Data Open Innovation Laboratory, AIST-Waseda University, 3-4-1 Okubo, Shinjuku-ku Tokyo, 169–0072 Japan

## Abstract

Massively parallel single-cell genome sequencing is required to further understand genetic diversities in complex biological systems. Whole genome amplification (WGA) is the first step for single-cell sequencing, but its throughput and accuracy are insufficient in conventional reaction platforms. Here, we introduce single droplet multiple displacement amplification (sd-MDA), a method that enables massively parallel amplification of single cell genomes while maintaining sequence accuracy and specificity. Tens of thousands of single cells are compartmentalized in millions of picoliter droplets and then subjected to lysis and WGA by passive droplet fusion in microfluidic channels. Because single cells are isolated in compartments, their genomes are amplified to saturation without contamination. This enables the high-throughput acquisition of contamination-free and cell specific sequence reads from single cells (21,000 single-cells/h), resulting in enhancement of the sequence data quality compared to conventional methods. This method allowed WGA of both single bacterial cells and human cancer cells. The obtained sequencing coverage rivals those of conventional techniques with superior sequence quality. In addition, we also demonstrate *de novo* assembly of uncultured soil bacteria and obtain draft genomes from single cell sequencing. This sd-MDA is promising for flexible and scalable use in single-cell sequencing.

## Introduction

Single-cell genomics enabled the exploration of cellular diversity in a broad range of biological samples^1, 2^. Nowadays, the use of this technique allows us to identify the genomes of uncultivable microorganism^3, 4^, genetic mosaicism in tissues^5^, and intra-tumor heterogeneity^6^, which brings new perspectives to our understanding by revealing the role of individual cells in the biology of complex ecosystems and organisms. However, we still face several technical challenges in the sample preparation process, including effective isolation and lysis of single cells, uniform amplification of whole genome, quality assessment of single-cell amplified genomes (SAGs), sequencing library preparation, and sequencing analysis. Among all, to maximize the quality and throughput of single-cell sequencing, there is a great demand for novel techniques, which enable massively parallel whole genome amplification (WGA) with high accuracy.

Microfluidic-based WGA represents one approach to achieve high-throughput and high fidelity single cell genomics. Microfluidic devices, including in-house^7–9^ and commercially available valve-controlled microfluidic circuit (Fluidigm C1)^10, 11^ and microwell^12, 13^, can integrate labor-intensive experimental WGA processes in a single, closed device and minimize the running cost and the risk of contamination that frequently occurs in bench-top experimentation. The reaction in microfluidic environment offers advantages over tube-based approaches, including improved reaction efficiency and detection sensitivity at the single-molecule level. In particular, droplet microfluidics has garnered the attention due to its scalability for various single cell studies^14^. Recently, we and other groups also developed the compartmented droplet multiple displacement amplification (cd-MDA) technique for bias-less single-cell WGA^15–18^. By distributing and amplifying single-cell genome fragments in 10^5^ droplets, we can obtain high quality SAGs as compared to conventional in-tube MDA. The microfluidic droplet is considered a suitable platform for handling single cells inside its closed environment and for processing cell contents^19^.

To accomplish the massively parallel single-cell WGA, consecutive reactions, which include processes from single-cell isolation to WGA, should be conducted in compartmented environments. Therefore, microchannel or microwell, which allows reagent addition or exchange easily, has been mainly used for multistep single cell reaction. However, the maximum number of reaction compartments is currently approximately 10^4^ due to the limitations of microfabrication and liquid control in parallel microchambers. On the other hand, droplet microfluidics provides a chemically closed reaction environment by emulsification. Although droplet-based WGA presents great advantages such as minimizing contamination risk and massive production of reaction environments, accurate and consecutive reagent addition into individual droplets must be provided for its use as a massively parallel single-cell genomics tool.

In this study, we developed a novel droplet-based WGA technique, which is equipped with the droplet-based single-cell encapsulation and subsequent reagent addition by one-to-one droplet fusion. This approach, which we call single droplet MDA (sd-MDA), enables massively parallel amplification of single cell genomes by high-speed generation of single-cell droplets and their passive fusion with MDA reagent droplets in microfluidic channels. The single cells encapsulated in each droplet were consecutively lysed. Their genomes were amplified individually and recovered as closed emulsion droplets in carrier oil without cross-contamination. Reduction of the reaction volume decreases the risk of encounter with environmental or reagent-borne contaminants and their unexpected amplification. We could obtain the SAGs with high coverage from both bacterial and mammalian cells encapsulated in droplets (about 10^6^ droplets/run). We then applied sd-MDA to a complex soil bacteria sample and obtained 17 draft genomes from single cell sequencing. Our results demonstrate the potential of sd-MDA as a tool for massively parallel single-cell genomics by increasing sample preparation efficiency, while reducing the cost and labor investment required for the investigation of genome diversity at the single-cell level, allowing the effective investigation of complete genomes of uncultured microbes collected from environmental samples and mutation analysis of tumor cells.

## Results

### Single droplet multiple displacement amplification (sd-MDA) workflow

**Figure 1:**
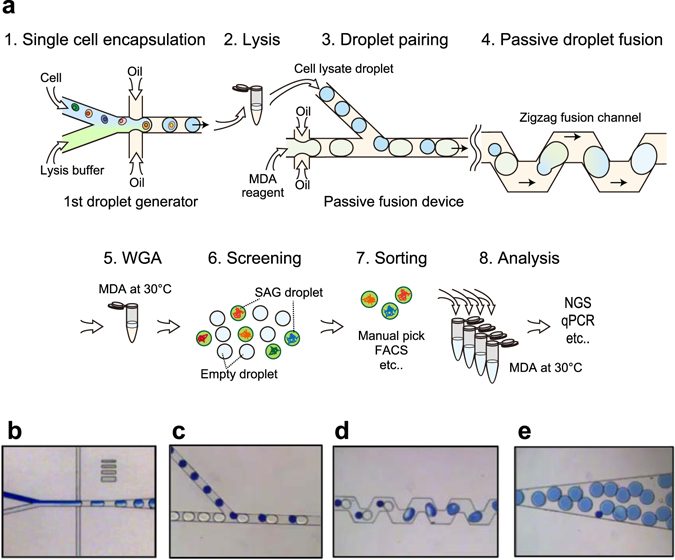
Massively parallel single-cell genome amplification by microfluidic droplets. (**a**) Workflow of sd-MDA and subsequent single cell genome analysis: (1) In the 1^st^ droplet generator, single cells were mixed with lysis buffer and immediately encapsulated into aqueous droplets in an oil-based emulsion using flow focusing. (2) Each cell was lysed within each droplet by off-chip incubation. (3) The cell lysate droplets were re-injected into the passive fusion device for pairing with the other droplets generated from MDA reaction mix reagent. (4) Paired droplets were serially fused together in the zigzag channel. (5) Single-cell whole genomes were individually amplified within droplets by sd-MDA reaction. (6) SAGs in droplets were screened with intercalating fluorescent dye. (7) Positive amplification droplets were individually sorted into microtubes by manual picking or FACS. (8) Sorted SAG droplets were re-amplified in tube for further analysis by NGS and qPCR. **(b**,**c**,**d** and **e**) Microphotographs of (**b**) the 1^st^ droplet generator, **(c)** droplet pairing channel, (**d**) following zigzag channel, and (**e**) downstream channel in the passive fusion device.

Our strategy was to use the droplets for compartmentalization of single cell and subsequent WGA (Fig. 1a). In this workflow, a number of single cells were first introduced into the droplet generator and compartmented in the droplets with cell lysis reagents (Fig. 1b). For the addition of WGA reagents to each droplet, massively produced droplets (10^5^ droplets) containing single cell lysates were re-injected into the droplet fusion device, which was modified from that reported by Mazutis *et al*.^20^ (Fig. 1c,d and Supplementary Movie S1, S2). A re-injected droplet containing single cell lysate was fused with a MDA reagent droplet only partially stabilized by surfactant, followed by stabilization of the newly formed droplet by additional surfactant injected into the zigzag channel against undesirable coalescence. The one-to-one fused droplets were directly collected from microchannels and incubated for single-cell MDA (Fig. 1e and Supplementary Movie S3). Thus, each single cell was independently and completely processed into SAGs in individual droplets. After 1^st^-round single-cell MDA reaction, the droplets containing SAGs were screened with DNA intercalating dye and isolated by manual picking or FACS. The droplets can be preserved in carrier oil for several weeks at 4 °C. For sequencing library preparation or other further analyses such as qPCR or target gene detection, the collected SAG droplets were re-amplified in a 2^nd^-round liquid MDA reaction.

### Droplet encapsulation of single cells and droplet fusion-based WGA

To demonstrate that our droplet system enables massively parallel generation of reaction environments for single-cell WGA, we first tested the performance with lab-cultured *E. coli* cells. Cell concentration was optimized under 0.1 cells/droplet to prevent the encapsulation of multiple cells in single droplets. In the droplet generator, monodispersed droplets with an average diameter of 40 ± 0.75 μm (volume: 34 pL) were generated at 21,000 droplets/min. The encapsulated cells were lysed in individual droplets prior to fusion with MDA droplets.

**Figure 2:**
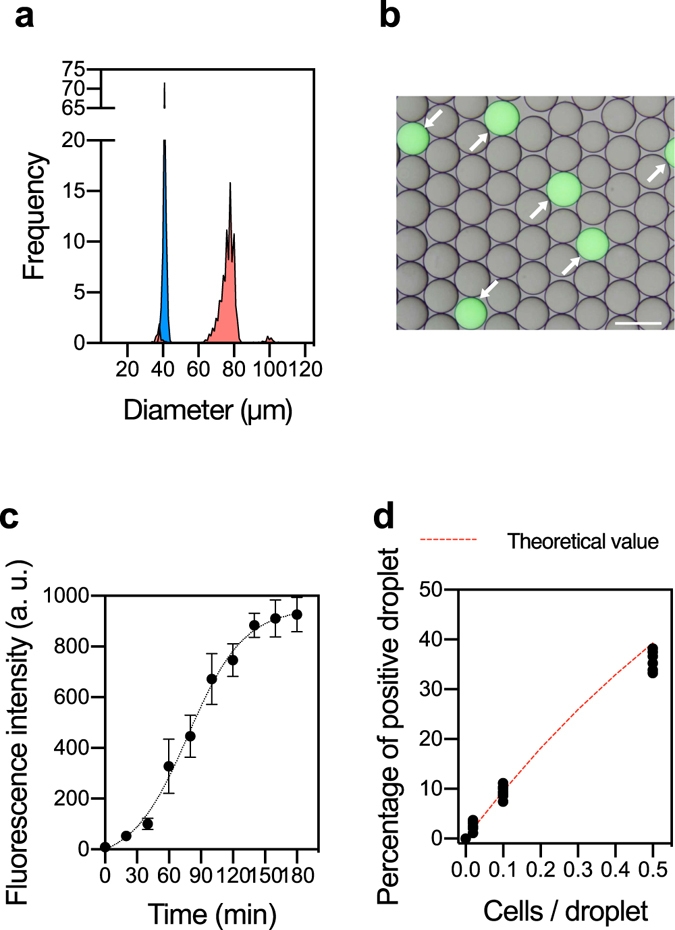
Droplet fusion and subsequent single-cell WGA in sd-MDA. (**a**) Histograms of droplets before (Cell lysate droplet: blue) and after fusion (SAG droplet: red). (**b**) Fluorescence image of droplets after the 1^st^-round MDA reaction. *E. coli* cells were introduced at 0.1 cells/droplet and their genomes were amplified for 2 h with Evagreen dye. Scale bar; 100 μm. **(c)** Time-dependent appearance of the fluorescence signal during amplification of single *E. coli* genome. All data are presented as averaged intensities of fluorescent positive droplets measured with SD, and 100 droplets were analyzed at each time point. (**d**) Relationship between introduced *E. coli* cell concentration and the number of fluorescent positive droplets.

Figure 2a shows the histogram of the diameter of droplet before and after fusion. After droplet fusion, the average diameter was 77 ± 2.94 μm (volume: 240 pL). Approximately 95.0% of single cell droplets were fused with MDA reagent droplet, while 3.0% of all droplets showed undesired coalescence. This result clearly indicated that the droplets fusion device could conduct passive and effective fusion. Almost all single cell lysate droplets were fused one-to-one with MDA droplets produced on-chip at <12,000 droplet/min. After off-chip isothermal MDA reaction from single *E. coli* cells, the appearance of fluorescent droplets was successfully observed (Fig. 2b), indicating the amplification of single cell genome within closed droplets. The fluorescence intensities of the droplets containing single cells gradually increased after 20 min and then reached a plateau after 180 min of incubation (Fig. 2c). Positive droplet rates followed the number of cells per droplet and correlated with the theoretical value (Fig. 2d), indicating that the single cells were successfully subjected to lysis and subsequent MDA within individual droplets. In the case of no cell sample, false fluorescent positive rate was less than 0.01%. To prevent the cross contamination, the cell concentration was optimized to about 1 cell to 10 droplets in the following experiments.

### Quality assessment of SAG obtained from sd-MDA

We evaluated the effect of prevention of contamination and amplification bias in single-cell WGA. We mixed *E. coli* and *B. subtilis* strains and encapsulated them in droplets. After the 1^st^-round MDA in the droplets, the sd-MDA products were estimated at approximately 100 pg DNA per positive droplet and subsequently re-amplified up to approximately 2 to 5 μg for further quality assessments and sequencing library preparation (10^9^-fold amplification). In the non-fluorescent droplets, assumed empty droplet, re-amplified products were less than 3 ng DNA. Hence, we could also identify positive and negative samples easily by endpoint amplicon yield.

For the quality assessment of SAGs obtained from single bacterial cells, 16S rRNA gene sequence was first analyzed in 60 SAGs re-amplified from randomly picked fluorescent positive droplets (n = 76), while other 16 droplet amplicons were omitted due to low amplicon yield equal to negative droplet for some reasons. The PCR amplicons of 16S rRNA gene were successfully observed from 56 of 60 SAGs and identified as either *E. coli* or *B. subtilis* sequences, while the other 4 samples showed no similarity to specific sequences.

**Figure 3:**
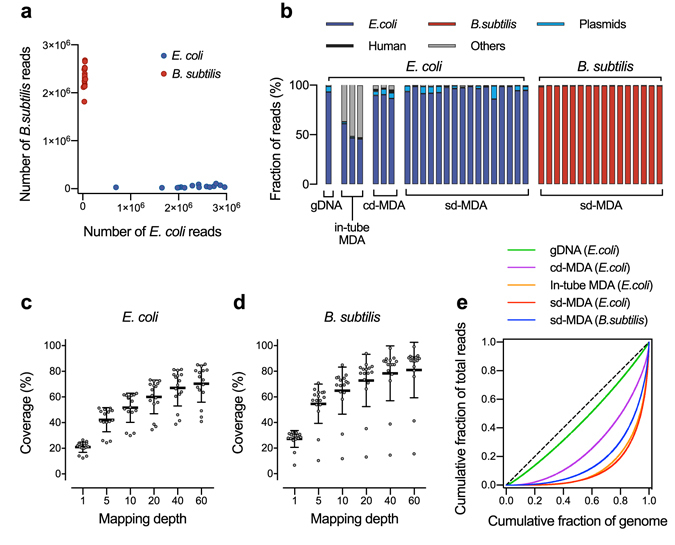
Evaluation of sd-MDA using species-mixing experiment. (**a**) sd-MDA analysis of mixtures of *E. coli* and *B. subtilis* cells. The scatter plot shows the number of reads mapped to *E. coli* and *B. subtilis* genomes associating to each droplet. Red dots indicate droplets that were identified from these 16S rRNA data as *E. coli*; Blue dots indicate droplets that were identified as *B. subtilis*. (**b**) Classification of raw sequence reads using Basic Local Alignment Search Tool (BLAST). (**c**,**d**) Genome coverage in *E. coli* (**c**) and *B. subtilis* (**d**) droplets. All samples were randomly down-sampled based on mapping depth to each genome. (**e**) Lorenz curves of coverage uniformity, showing the relationships between the cumulative fractions of the genome covered (x-axis) and the cumulative fraction of mapped reads (y-axis). Each curve show averaged data, excepting outliners. Data were compared with gDNA and single-cell *E. coli* data produced using conventional in-tube MDA reactions and cd-MDA reaction^16^.

To further confirm the accuracy of sd-MDA product, we analyzed the rate of sequence read mapped to both *E. coli* and *B. subtilis*. To obtain high-depth sequence data in the same sequencing condition, we randomly selected 32 (identified as *E. coli* (n = 16) and *B. subtilis* (n = 16) based on 16S rRNA gene identification) samples out of 56 SAGs and sequenced them together. If each droplet product represents either *E. coli* or *B. subtilis* single cell, its corresponding sequence reads should map overwhelmingly to either the *E. coli* or *B. subtilis* genome. Indeed, we observed that all of 32 sequenced SAGs had >94% of their reads mapping to *E. coli* or *B. subtilis* (Fig. 3a and Supplementary Table S1). The droplet-derived SAGs showed strong enrichment for reads mapping to the reference genome expected from 16S rRNA gene identification, and cross-mapping rates were comparable with those pre-purified gDNA samples. The lack of cross-contamination in the positive droplet samples indicated that sd-MDA could be independently conducted against single cells encapsulated in single droplet reaction vessels. In addition, as shown in Fig. 3b, sd-MDA showed a significant decrease of unexpected contamination reads, which were mapped to human genome or were unmapped to reference genomes, in *E. coli* (1.0%) and *B. subtilis* (0.6%) SAGs compared to conventional in-tube MDA (47.3%) and our previous cd-MDA (6.0%)^16^. Major unmapped reads from sd-MDA in both *E. coli* and *B. subtilis* had no identifiable sequence and attributed artificial product, except for the unmapped reads from *E. coli*, which classified as f plasmid and phage lambda contained in *E. coli* K-12 genome (3.7%) (Fig. 3b). This result indicated that the unexpected amplification of contaminating DNA (human or other organism-derived contaminants) was highly suppressed in sd-MDA, resulting in the enrichment of SAG from single cell in single droplet. Meanwhile, from the low yield 2^nd^-round MDA samples and samples with no similarity to specific 16S rRNA gene sequences, we could obtain a small number of sequence reads (less than 30,000 reads) insufficient to cover the whole genome, even though they were overwhelmingly mapped on either *E. coli* or *B. subtilis* genomes. Thus, the QC based on the 2^nd^-round MDA yield and 16S rRNA gene identification would be useful for the screening of sequencing library.

Next, we evaluated coverage breadth of sd-MDA products from single model bacteria. At 60× mean mapping depth, raw sequence reads covered averages of 70.4 ± 14.0% of genome in *E. coli* cells (Fig. 3c) and 80.9 ± 21.0% of genome in *B. subtilis* cells (Fig. 3d). The superior coverage performance in *B. subtilis* may be attributable to the lower GC content and genome size of *B. subtilis* (43.9%, 4.0 Mbp) compared with those of *E. coli* (50.8%, 4.6 Mbp) and better accessibility of the genome after lysis^21^. The coverage values for each cell are in line with typical single-microbe genome sequencing with in-tube MDA^11, 16, 22^. Several outliners, which showed low coverage compared to the others, were observed in both *E. coli* and *B. subtilis*. These samples contained partially amplified genome as compared to other samples.

To evaluate the coverage uniformity of sd-MDA, we compared the Lorenz curve of sd-MDA with conventional tube MDA and cd-MDA^16^. A shown in Fig. 3e, the bias in sd-MDA product was comparable with conventional tube method, though the reduction of WGA reaction volume may prevent amplification bias according to previous reports^8, 12^. In addition, the number of chimeric reads in sd-MDA was slightly lower than that in the conventional in-tube MDA (Supplementary Table S1). Regarding the amplification bias suppression and suppression of chimera reads, our cd-MDA technique outperformed both conventional in-tube and sd-MDA reactions.

### *De novo* assembly of SAG obtained from sd-MDA

**Figure 4:**
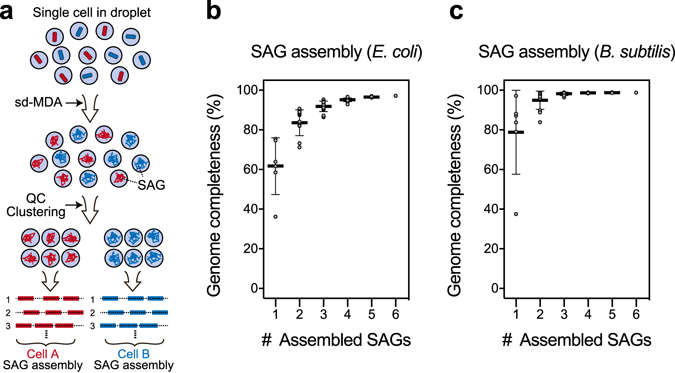
Integration and *de novo* assembly of SAGs obtained from sd-MDA. (**a**) SAG assembly from sequence data obtained from each sd-MDA product based on marker gene such as 16S rRNA sequence. Percentage of the genome recovered in the assembly when the reads from multiple SAGs are combined together and assembled. (**b**) *E. coli* and (**c**) *B. subtilis*.

Next, we assessed the quality of *de novo* assembly of sequence reads obtained from sd-MDA products. As shown in Supplementary Table S2, similar to the raw read mapped rate, coverage of *E. coli* single cell was 63% and less than previous cd-MDA technique (90%). This result was attributed to its lower uniformity of SAG as compared to cd-MDA and several outliers. However, in the sd-MDA, we can generate massive SAGs using droplets in a parallel manner. Thus, we can integrate SAGs derived from identical cell types and perform *de novo* assembly to improve the genome recovery from identical cells (Fig. 4a)^23^. In this experiment, the sequence reads of SAGs showing either *E. coli* or *B. subtilis* 16S rRNA sequence were integrated and assembled *de novo*, resulting in integration of more than 3 SAGs covering approximately 90% of the genome (Fig. 4b,c). In addition, the contamination rate of these assembled data was evaluated by CheckM^24^. In sd-MDA, contaminated assembled contigs (0.42%) and fully unaligned contigs (4.23%) were significantly decreased compared to those detected with previous methods (3.2% and 39.8% in cd-MDA and 28% and 72% in tube MDA, respectively) (Supplementary Table S2 and Supplementary Fig. S1). Thus, the contigs of sd-MDA were highly enriched with single cell encapsulated in single droplet.

### Single cell sequencing of cancer cells

We also evaluated the application of sd-MDA for human cancer cell lines, which present a large genome size as compared to bacteria. Cellular nuclei were prepared and introduced into droplets for the 1^st^-round sd-MDA. Lysis and subsequent WGA in the droplets could be observed as spreading of DNA intercalating dye, which was used for nuclear stain in advance. Few incompletely lysed nuclei were observed, which retained their morphology inside droplets (Supplementary method). In comparison with previous work, single nuclei of SK-BR-3 with full-set of chromosome loci, which was assessed by a PCR-based quality assay (Supplementary Fig. S2)^25^, were selected for subsequent library construction and next-generation sequencing. Two single nuclei samples achieved high coverage depth (20×, n = 2) and breadth (93%, n = 2) comparable with a recent method for single cancer cell sequencing (Supplementary Table S3). In addition, over 99.5% of sequence reads were mapped to the human reference genome, indicating the lack of unexpected sequence reads derived from contaminants. Next, we calculated error rates, including the allelic dropout rate (ADO) and false positive rate (FPR) by comparing single cell variants to the population data^26^. Our analysis suggested that the ADO (19.2 and 28.7%) and FPR (4.6 × 10^−5^ and 5.2 × 10^−5^) were also comparable to those described in previous reports^15, 25, 26^. For coverage uniformity, we generated the Lorenz curve (Supplementary Fig. S3) and compared Gini’s coefficient of sd-MDA with previous studies. We found that the Gini’s coefficient was comparable to Nuc-seq^26^, which is reported as a high-coverage, whole genome and exome single cell sequencing method (0.57 in sd-MDA and 0.55 in Nuc-seq).

### Application for SAG collection from soil bacteria

**Figure 5:**
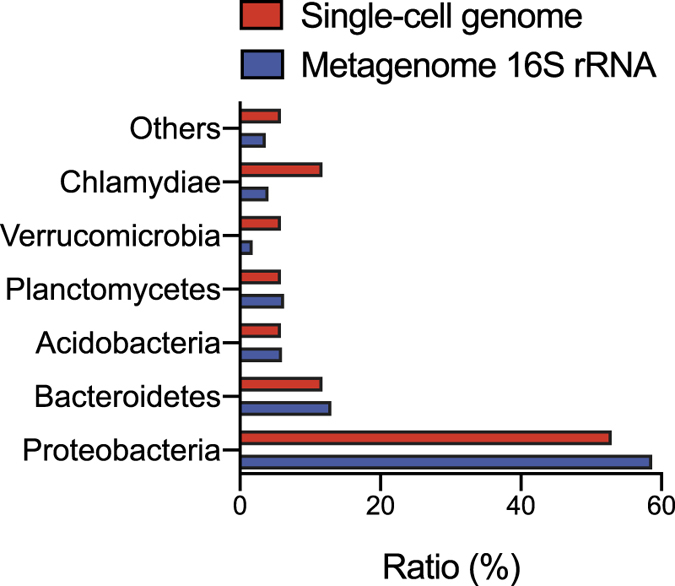
Comparison of soil microbial phyla distribution from single-cell assemblies and metagenome 16S rRNA gene sequence data. *De novo* assemblies were performed from single-cell sequencing data obtained from sd-MDA-derived 17 SAGs.

For this application, single-cell genome sequencing of soil samples was performed using sd-MDA. The soil sample contained a vast uncharacterized diversity of microorganisms and presented the inherent spatial heterogeneities. Although a recent metagenomics approach characterized communities on the basis of the relative abundance of genes, genome assembly from individual genes is difficult, owing to chimeras with undefined similarity. In contrast, single-cell genome sequencing has the potential to resolve this problem, but conventional techniques yield only partial genomes from limited samples. Thus, we attempted to obtain high completeness genomes from single soil bacteria using sd-MDA. We picked 48 fluorescent positive droplets after the 1^st^-round amplification. After the screening of the 2^nd^-round MDA product, 37 samples (77%) showed sufficient amount of DNA, resulting in the same successive rate as the model bacteria. In the 16S rRNA gene sequence analysis, no amplicon was obtained from 11 out of 37 samples, while the amplicons from 17 out of 37 samples were identified as bacterial 16S rRNA genes. Then, for assessment of single-cell sequence data that included unknown uncultured bacteria, we carried out *de novo* assembly of the single-cell data sets and assigned taxonomy by 31 single-copy bacterial marker genes at the family level by AMPHORA2. We classified all 17 assemblies as SAGs (size range: 0.4–4.6 Mbp) and strong enrichment of sequences from a single taxonomy (Supplementary Table S4). In addition, 14 of 17 assembled SAGs were assigned to any OTU defined by 16S rRNA gene sequence in metagenomic analysis, indicating that SAGs were certainly derived from the soil sample. The proportion of detected organisms of SAGs was compared that of soil metagenome 16S rRNA gene analysis. Both approaches recovered the same top microbial phyla (Fig. 5). The estimation of genome completeness and contamination suggested that most SAGs obtained from sd-MDA had relatively high completeness (approximately 50–80%) with low contaminants (<5.2%). This result correlated with the performance on genome assembly from model bacteria samples. However, several SAGs showed relatively low genome completeness (<20%) or/and high contamination percentage (20–50%) or multiple taxonomies identified by marker genes. These differences could be attributed to the difficulty in single cell preparation from soil aggregates, cell lysis, and subsequent nucleic acid extraction from soil sample, which presents unknown impure substances and inhibitors.

## Discussion

Single cell sequencing requires the unbiased WGA without contamination from tiny amounts of single cell genome in a high-throughput manner. The interrogation of thousands of single cells is required to address many of the fundamental biological questions with single-cell analysis^1, 27, 28^. Supplementary Table S5 shows the comparison of our sd-MDA with conventional single-cell WGA platforms. FACS-associated multi-well platform has been widely used for single-cell isolation and subsequent reactions, including cell lysis, WGA, QC, and library preparation^25, 29, 30^. In order to add various reagents to each reaction vessel at each reaction point, the automated liquid handling system or robotics has been frequently used to minimize the labor required for hundreds of amplification reactions and unexpected contamination^31^. However, this approach requires a large machine and significant running cost, and its throughput is limited by the well numbers (up to about 10^3^ wells/plate) in the plate and liquid control system. Another commercially available system for single cell genomics is Fluidigm C1, an automated the process of single cell isolation, lysis, and WGA, but the system only targets mammalian cells and its throughput is about 96 cells per 9.5 h of running (approximately 10 single-cells/h). On the other hand, droplet microfluidics can process tens of thousands of single cells in parallel^19, 32^. In this study, we designed sd-MDA as >10^5^ 240 pL-reactions per microfluidic circuit running in two microfluidic devices to handle both bacterial cells and mammalian cells in the same condition. The sd-MDA avoids the possibility of cross-contamination between reactions by preventing droplet coalescence with surfactant contained in carrier oil and minimizes unexpected amplification due to its small reaction volume. Because the droplet reactions, including single cell isolation, lysis, and WGA, are conducted entirely and only by introducing the reagents into disposable single-use devices, the cost, labor, and time are significantly reduced (0.02¢ per cell). Throughput of sd-MDA is estimated as 84,000 cells per 4 h of running (21,000 single-cells/h). These performances strongly indicated that our system has great potential for flexible and scalable use in single cell analysis.

In this study, we developed one-to-one fusion of polydispersed droplets in the microfluidic device to add the enzyme mix after cell lysis with alkaline treatment. We used a passive droplet fusion device, which does not require any electric fields, lasers, or specific channel treatment to induce droplet fusion^20^. The device is therefore simple to use and manufacture, requiring no electrode or surface patterning. In contrast to other mixing techniques using picoinjection^33^, we could combine large droplets that can encapsulate single or more mammalian cells or bacterial cells. This zig-zag fusion approach could be applicable for multi-step reactions by repetitive droplet fusions since we can operate droplets in a broad size scale between picoliter and nanoliter^16^. In order to handle various sizes of droplets or multiple fusions, physical or chemical enhancement of droplet fusion would be helpful^34^.

In general, the cellular biological state has a major influence on the genome amplification performance^25, 31^. For example, single cells, which are sorted by excluding apoptotic cells and cellular debris or by purifying polyploid cells, exhibit lesser genome amplification bias than unsorted single cells^22, 25, 26^. In addition, in the case of environmental microbial samples, additional sample preparation and considerations of single-cell sorting conditions are also required to obtain SAGs efficiently^30^. In our experiment using a bacterial cell mixture as the model sample, we analyzed 60 SAGs from sd-MDA samples. In this experiment, cells were not FACS-sorted and were directly introduced into droplet within the microchannel. Thus, we considered that several outliners, which showed low coverages compared to the others, were attributed to apoptotic cells, cellular debris, or incompletely lysed cells, for which reduced amplification uniformity relative to intact and viable cells would be expected. In the sd-MDA, because the starting reaction environment is quite small, contaminants carried from reagents and sample could be effectively reduced compared to the previous methods^13^. Although our previous cd-MDA utilized droplets to distribute single-cell genome fragments into multiple droplets^16^, the undesired contaminants were also amplified, resulting in unmapped raw sequence reads. These minor unmapped raw reads could cause a number of contaminated contigs, which were unaligned to reference genomes (Supplementary Fig. S1). In contrast, sd-MDA dramatically decreased contaminant reads and contigs as compared to cd-MDA and conventional in-tube MDA.

However, further minimization of amplification bias should be considered in sd-MDA. Although several studies reported that the MDA in nanoliter volumes could prevent the amplification bias^8, 12^, our amplicon obtained from picoliter droplet in sd-MDA showed bias comparable to conventional tube reaction. In addition, there is a discrepancy in the effect of genome amplification time on bias and coverage between previous reports^25, 31^. Thus, we considered that the MDA conditions such as reaction time and reagent components for sd-MDA should be carefully optimized for target cell properties to reduce the bias and obtain more genome coverage from single cells.

The other technical limitation of sd-MDA is the requirement for a 2^nd^-round amplification for sequencing analysis, which generally requires nanograms of template DNA in each library preparation. In this study, fluorescent positive droplets were isolated by manual picking under clean booth after the 1^st^-round MDA reaction. Although the use of FACS with double emulsion droplets would enhance the throughput of single cell sequencing^35^, the additional instrumental setup and double emulsification of droplets could cause undesired contamination and decrease the effectiveness. Therefore, we realized that we have to integrate the DNA barcoding with sd-MDA for massively parallel and non-labor intensive single-cell genome sequencing^19^. To achieve this, we must operate repetitive droplet fusions for conducting multistep reactions from cell isolation to SAG barcoding and optimize various conditions such as sample cell number, barcode number, and sequencing depth according to the capacity of sequencer and research targets. We think that the single-molecule droplet barcoding strategy^36^ will suit our fusion system compared to the barcoding bead strategy^19^, which requires a higher flow rate to carry beads. Furthermore, the integration of a multi-step reaction, including barcoding with sd-MDA, would be useful for various sequencing analyses of SAGs such as low-pass whole genome sequencing^37^ and target sequencing, including epigenome studies such as Assay for Transposase-Accessible Chromatin (ATAC)-seq^38^.

Reconstruction of each cell’s genome within a complex microbial community is one of the goals for microbial ecology and environmental microbiology. In general, single-cell sequencing of uncultured environmental bacteria has some technical challenges and SAG recovery rate is highly variable depending on the nature of the sample. First, because of the difficulty in FACS isolation of environmental bacteria, only 2–3% of the reaction sample showed positive amplification in 16S rRNA gene screening^3^. Second, the contaminated sequence could interfere with genome assembling from SAGs, resulting in misinterpretation of the uncultured bacterial genome^39, 40^. Thus, genome recovery rate is still low because of the amplification bias and formation of chimeras in a conventional reaction format. In fact, the completeness of the recovered SAG was 40–55% and the success rates for soil samples tend to be low (<10%)^30^. However, in sd-MDA, single cells were randomly encapsulated within droplets and then they were subjected into WGA in a simple microfluidic flow. The droplets containing positive WGA reaction could be screened with a DNA intercalating dye, with increased hit rate of 16S rRNA gene screening (35%, 17 of 48 samples). Owing to these advantages, the completeness and contamination rate were improved to a relatively high level (approximately 50–80%). Moreover, contamination-free contigs obtained by sd-MDA would be useful for genome assembly from unknown bacterial cell sample with the prevention of misassembling and chimeric formation between contaminants. Overall, our single-cell analysis of soil bacteria demonstrated the applicability of the sd-MDA to the analysis of microbial diversity in a real-world sample with the genome assembly from single-cell sequencing. In this study, a small number of droplets (48 droplets) were randomly picked from 10^5^ positive droplets and the quality of each amplicon was assessed by sequencing. The assembled single-cell genomes obtained from each SAG showed the diversity of soil bacteria and certainly presented their proportion based on the analysis of 16S rRNA gene sequences of soil metagenome. As compared to lab-cultured model bacteria samples, the SAG obtaining rates and their estimated qualities (i.e., completeness and purity) are slightly low due to the difficulty of sample preparation and various impurities in real samples. Indeed, the cell preparation from environmental samples, such as lysis condition, influences the success of enrichment of SAGs and should be carefully considered in accordance with the sample itself. However, as sd-MDA potentially allows the acquisition of contamination-free SAGs in a massive and parallel manner, co-assembling of multiple SAGs based on the identity of putative taxonomy would help compensating the weakness of low coverage and increase completeness of the genome assembly. The recovery of partial genomes from single cells is due to parts of the genome being significantly over amplified compared to other regions. As the amplification bias occurs randomly in MDA, different SAGs from the same strain should recover different portions of the overall genome, thus complementing each other when being co-assembled. Indeed, our co-assembled results (Fig. 4) demonstrated that combined assemblies allowed the recovery of more complete genomes and was consistent with previous results^23^. This analysis procedure would be useful for obtaining near-complete or complete bacterial genomes from single cells in environmental samples^3, 41^.

On the other hand, single-cell sequencing is becoming a valuable tool for dissecting intra-tumor genetic heterogeneity at the single-cell resolution when investigating cancer development and evolution^1, 28^. In this study, sd-MDA showed higher coverage and comparable performance in ADO and FPR compared to the previous techniques applied for the study of tumor genetic heterogeneity. The sd-MDA-based sequencing approach on individual tumor cells can provide accurate sequences of cancer-associated mutations such as single nucleotide variation (SNV), in a massive and parallel manner. Cost and throughput of single cell sequencing experiments have been major barriers in cancer genomics and analysis of genetic mosaicism in multicellular organisms. To address this, by increasing the throughput via combining barcoding and pooling approaches, this technique would provide new insights for cell-to-cell genomic differences in complex multicellular systems and the role of rare cells in population. In conclusion, we established the sd-MDA and demonstrated its capacity to analyze biologically important samples, including both mammalian and bacterial cells. Based on our findings, we propose that sd-MDA is one of the most practical supporting approaches for single-cell genome sequencing analyses.

## Methods

### Model bacteria preparation

For sequencing analysis of single microbial cells, *E. coli* K-12 strain (ATCC 10798, genome size: 4.6 Mbp) and *B. subtilis* (ATCC 6633, genome size: 4.0 Mbp) were used. *E. coli* K-12 cells were pre-cultured in Luria-Bertani (LB) medium (1.0% Bacto-tryptone, 0.5% yeast extract, 1.0% NaCl, pH 7.0) for 16 h and collected by centrifugation. *B. subtilis* cells were pre-cultured in Brain Heart Infusion Broth (ATCC medium 44, Thermo Fisher Scientific, San Jose, CA, USA) for 16 h and collected by centrifugation. The collected cells were washed three times with UV-treated Dulbecco’s Phosphate-Buffered Saline (−) (DPBS, Thermo Fisher Scientific). The cell concentrations were adjusted to 1.2 × 10^3^–3.0 × 10^4^ cells/μL for encapsulation into droplets at the concentration of 0.02–0.5 cells/droplet. All preparations for the cell suspension and reaction mix described below were performed under open clean bench KOACH 500-F (KOKEN LTD., Tokyo, Japan).

### Environmental bacteria sample preparation

Soil samples were collected beneath zelkova trees (Shinjuku-ku, Tokyo, Japan). Soil metagenomic DNA was isolated and purified from 1.5 g of soil sample by using ISOIL (Nippon Gene; Tokyo, Japan) according to the manufacturer’s protocol. For isolating bacterial fractions, 1.5 g of soil samples were suspended to 3 mL of DPBS(−). After vortexing thoroughly, the mixture was left to stand for 5 min. We collected the supernatant and centrifuged at 200 × *g* for 5 min. The supernatant was collected and filtered through a cell-strainer cap with a 35-μm nylon mesh (Corning, NY, USA). After the filtration, the cell suspension was centrifuged at 13,000 × *g* for 20 min. The pellet was washed twice with DPBS(−) before droplets generation.

### Mammalian cell preparation

For sequencing analysis of single cancer cells, SK-BR-3 cell (ATCC HTB-30, Manassas, VA, USA) and HCT116 cell (ATCC CCL-247) were used. SK-BR-3 cells were pre-cultured in McCoy’s 5a (Modified) Medium (Thermo Fisher Scientific) for 3 days, and collected by centrifugation. HCT116 cells were pre-cultured in Dulbecco’s Modified Eagle Medium (DMEM, Thermo Fisher Scientific) for 3 days, and collected by centrifugation. The collected cells were washed three times with DPBS(−) with UV treatment. Then, cell suspensions were treated with NST-DAPI buffer for 15 min for nuclei preparation^25^. The nuclear suspensions were filtered through a cell-strainer cap with 35-μm nylon mesh, and washed twice with DPBS(−). The nuclear concentration was adjusted to 6.0 × 10^3^ cells/μL for encapsulation into droplets.

### Fabrication of a microfluidic droplet generator and droplet fusion device

Two types of microfluidic devices were designed using AutoCAD (AutoDesk, Sausalito, CA, USA), and fabricated using conventional soft lithography techniques according to previous reports^16, 32^. Photomask patterns were transferred to a layer of negative photoresist (SU8-3050, Microchem, Newton, MA, USA) coated on a glass wafer (40 mm × 49 mm), and master molds were fabricated. In the 1^st^ droplet generator, microchannels were 50-μm tall and 100-μm wide, except at the cross-junction area. The cross-junction was designed to be 34-μm wide for the aqueous phase and 20-μm wide for the continuous oil phase. In the droplet fusion device, microchannels were 50-μm tall. The device was designed according to a previously reported droplet fusion device, with some modifications^20^. The microfluidic device consisted of several sections such as sections containing droplet re-injection, on-chip droplet generation, droplet pairing, droplet fusion, and droplet collection. Droplets were coalesced at the fusion module containing zigzag channels. Poly(dimethylsiloxane) (PDMS; Sylgard 184: Dow Corning Corp., Midland, MI, USA) and its cross-linker were mixed thoroughly at a ratio of 10:1 (w/w) and then degassed. The PDMS mixtures were poured over the master molds and cured for at least 2 h at 70 °C. After curing, the PDMS slabs were carefully peeled off the molds, and the slabs were punched with a 0.75-mm biopsy punch (World Precision Instruments, Sarasota, FL, USA) for connection to pumps via PTFE tubing (AWG 24). The punched PDMS slabs and PDMS-coated glass slides were bonded by plasma treatment (Plasma Cleaner PDG-32G, Harrick Scientific, Ossining, NY), followed by baking for at least 30 min at 70 °C. Finally, to produce a hydrophobic surface coating, the microchannel was filled with Aquapel solution (PPG Industries, Pittsburgh, PA), and then excess Aquapel was blown off with air.

### Microfluidic droplet manipulation for single-cell encapsulation by the droplet generator

In the 1^st^ droplet generator, cell suspension and buffer D2 reagent (QIAGEN, Hilden, Germany) for cell lysis were pumped from different two inlets into the cross-junction as dispersed-phase liquids, while the carrier phase fluorinated oil HFE7500 containing 2% (v/v) of the surfactant Pico-Surf1 (Dolomite, Charleston, MA, USA) was driven from the other inlet using Mitos P-Pump (Dolomite). The cell suspension and lysis reagent met at the cross-junction, and droplets were periodically pinched off from the dispersed phase, at flow rates of 24 μL/h for both dispersed-phase liquids and 360 μL/h for the carrier oil. The device outlet was also connected to a collecting PCR tube via PTFE tubing. The total 10–40 μL of cell aqueous solutions were converted into approximately 0.3–1.2 × 10^6^ droplets (diameter: 40 μm). The cells encapsulated into individual droplets were then incubated at 65 °C in PCR tubes for 10 min.

### Droplet fusion and single-cell whole genome amplification

Prior to reagent introduction into the device, an MDA mixture (REPLI-g Single Cell Kit, QIAGEN) was prepared containing 1.5 μL of stop buffer, 1.5 μL of H_2_O, 14.5 μL of reaction buffer, 1.0 μL of enzyme mix, 2.5 μL of 10% Tween-20 (1% v/v concentration), and 0.5 μL of 20x Evagreen (Biotium, Fremont, CA, USA), for use in a 21.5-μL reaction volume. The MDA mixture was mixed gently, but completely, by vortexing and loaded into the microfluidic device. For cell lysate droplet prepared above, the surrounding carrier oil was replaced by FC-40 containing 2.0% (v/v) Pico-Surf1 (Dolomite). By using tubes and pumps as mentioned above, the droplets were then re-injected into a microfluidic device where they were spaced by FC-40 oil and fused one-to-one with MDA droplets (diameter: 73 μm) produced on-chip with FC-40 oil containing 0.02% (v/v) Pico-Surf1. The fused droplets (diameter: 77 μm) were stabilized in zigzag channels with FC-40 oil containing 2.0% (v/v) Pico-Surf1 introduced from the other inlet. After collection in PCR tubes, the fused droplets were washed with HFE7500 containing 2.0% (v/v) Pico-Surf1 and then incubated at 30 °C in PCR tubes for 2 h. Droplets were then transferred onto glass slide for microscopic observation and droplet picking. Fluorescent positive droplets were manually collected by using a micropipette under the open clean system. Each droplet was individually transferred into microtubes containing reagent for a 2^nd^-round MDA reaction. After 2 h of incubation at 30 °C, the enzyme was inactivated at 65 °C for 3 min. In cell encapsulation and whole genome amplification, we applied the same protocol for both bacterial cells and human cancer cells. Both kinds of cells were encapsulated in droplets without any modifications of flow conditions. In addition, though we confirmed that other commercially available whole genome amplification kits were applicable for our system, we used REPLI-g Single Cell Kit in this study because it was optimized to alkaline lysis condition and easily incorporated into the system toward both use of mammalian cells and bacterial cells.

### DNA quantification and PCR-based quality assay

For the quantification of DNA yield after the 1^st^-round MDA of single *E. coli* cell sample, fluorescent positive droplets were individually picked and transferred into MDA mixture with Evagreen. Then, the 2^nd^-round MDA was conducted and the increase of fluorescence intensities was monitored by using StepOnePlus Real-Time PCR System (Thermo Fisher Scientific). The DNA yield of each droplet was calculated compared with the Ct value of standard *E. coli* gDNA samples. The amplicon yields of the 2^nd^-round MDA were quantified by Qubit dsDNA HS assay kit (Thermo Fisher Scientific). The PCR-based quality assay was performed to assess the amplification bias in mammalian cell amplicons. For human cancer cells, 22 different single-copy loci were chosen from each chromosome of human genome, and the Ct value was calculated using SYBR assays (PowerUp SYBR Green Master Mix, Thermo Fisher Scientific)^25^.

### 16S rRNA gene sequence analysis for bacterial cells

In order to confirm amplification from single bacteria, 16S rRNA gene identification was also performed against SAGs obtained after the 2^nd^-round MDA. Primer pair sequences for the V3 and V4 region were used according to the protocol of Illumina Miseq system for 16S metagenomics sequencing library preparation. The PCR amplicons of 16S rRNA gene fragments (V3-V4 region) were sequenced by MiSeq (Illumina, San Diego, CA, USA) according to the Illumina protocol. For 16S analysis of soil metagenome, RDP classifier 2.11 was used for taxonomic assignments.

### Library preparation and whole genome sequencing

For the sequencing analysis, Illumina libraries were prepared using amplicons from the 2^nd^-round reaction of sd-MDA. In the case of model bacterial cells and soil sample, the Illumina library was prepared using Nextera XT DNA sample prep kit (Illumina) or QIASeq FX DNA Library kit (QIAGEN) according to the manufacturer’s instructions. Each single bacterial cell library was sequenced on an Illumina Miseq instrument using 2 × 300 paired-end reads. In the case of human cancer cells, the Illumina library was prepared using TruSeq DNA PCR-Free Library Preparation Kit and sequenced on Hiseq 4000.

### Sequencing analysis

For bacteria analysis, acquired reads were normalized to 1×, 5×, 10×, 20×, 40×, and 60× coverage per each genome for each sample. All sequence data were mapped to the NCBI reference genome of NC_00913 (*E. coli* substrain MG1655) or NCBI reference genome of NC_014479 (*Bacillus subtilis* subsp. *spizizenii* str. W23) using the software BWA^42^. Genome coverage was calculated using SAMtools^43^. Each normalized read was assembled *de novo* using SPAdes 3.5.0^44^, and the contigs were qualified by QUAST 2.3^45^. CheckM 1.0.6^24^ was used to assess the completeness and the contamination read rate after contigs smaller than 2,000 bp were removed. For SAG assembly, reads from single *E. coli* cells or *B. subtilis* were subsampled to 60× coverage per genome. These subsampled reads were combined together and assembled with SPAdes.

For cancer cell analysis, acquired reads were mapped to the UCSC human genome 19 (hg19) using BWA. After removal of reads with mapping quality score less than 40 from created SAM files, genome coverage was calculated using SAMtools. The Genome Analysis Toolkit (GATK)^46^ was used to detect single nucleotide variants (SNVs) of single cancer cells and population data (NCBI SRA053195)^26^. The GATK UnifiedGenotyper was used to generate a single Variant Call Format file from all single cells and population data. Detected variants were recalibrated using the GATK VariantRecalibrator and database constructed from hapmap 3.3, dbSNP build 138, Omni 2.5 M chip, and Mills. Using recalibrated SNVs, allelic dropout rate (ADR) and false positive rate (FPR) were calculated.

### Accession number

The sequence data for single cells amplified with the sd-MDA have been deposited in DNA Data Bank of Japan (DDBJ) under the accession number DRA005326. The sequence data for soil metagenome have been deposited in DNA Data Bank of Japan (DDBJ) under the accession number of DRA005327.

## Electronic supplementary material

Supplementary information

Supplementary movie 1

Supplementary movie 2

Supplementary movie 3
