# Supplementary material for: Massively parallel whole genome amplification for single-cell sequencing using droplet microfluidics
Source: Sci Rep. 2017 Jul 12;7:5199. doi: 10.1038/s41598-017-05436-4 (PMC5507899; doi:10.1038/s41598-017-05436-4)
Supplement: Supplementary file 1 — Supplementary information [file 41598_2017_5436_MOESM1_ESM.docx]

**Supplementary Information: Massively parallel whole genome amplification for single-cell sequencing using droplet microfluidics**

Masahito Hosokawa^1,2^, Yohei Nishikawa^3^,^,^ Masato Kogawa^3,4,^ and Haruko Takeyama^1,3,4^*

^1^ Research Organization for Nano & Life Innovation, Waseda University, 513 Wasedatsurumaki-cho, Shinjuku-ku, Tokyo 162–0041, Japan

^2^ PRESTO, Japan Science and Technology Agency (JST), 5-3 Yonban-cho, Chiyoda-ku, Tokyo 102–0075, Japan

^3^ Department of Life Science and Medical Bioscience, Waseda University, 2-2 Wakamatsu-cho, Shinjuku-ku, Tokyo 162–8480, Japan

^4^ Computational Bio Big-Data Open Innovation Laboratory, AIST-Waseda University, 3-4-1 Okubo, Shinjuku-ku, Tokyo 169–0072, Japan

Correspondence and requests for materials should be addressed to H.T.(email: haruko-takeyama@waseda.jp)

**Supplementary methods**

**Droplet monitoring**

Droplet generation and fusion were monitored with a microscope (CKX41; Olympus Corporation, Tokyo, Japan) and high-speed camera (FHS-33, FLOVEL CO., LTD, Tokyo, Japan). For monitoring the MDA, droplets were then transferred into capillary tubes (VitroCom, Mountain Lakes, NJ, USA) for microscopic observation. Bright-field and fluorescent images were captured every 20 min using a fluorescence microscope (BX51; Olympus Corporation) integrated with a digital camera (DP-73; Olympus Corporation). The diameter of the generated droplets and their fluorescence intensities were calculated using ImageJ software (http://rsb.info.nih.gov/ij). Hundred droplets were analyzed to acquire the average intensity of fluorescent positive droplets at each time point. For monitoring the efficiency of human cancer cell lysis, cells were treated with NST-DAPI buffer before encapsulation. We observed the morphological change of the nuclei inside the droplets. When the nuclei were lysed completely, the fluorescence signal derived from DAPI spread inside the droplets after DNA amplification. However, when lysed incompletely, the nuclei stained with DAPI maintained their morphology. We confirmed that almost all the nuclei were completely lysed after the first round of amplification.

**Supplementary Figure S1**. Classification of assembled contigs using Basic Local Alignment Search Tool (BLAST).

**Supplementary Figure S2**. Quality control assay using a panel of 22 chromosome-specific PCR primers to determine the sd-MDA amplification efficiency of each single nucleus. The numbers of inserted in each cell are Ct value of SYBR assay.

**
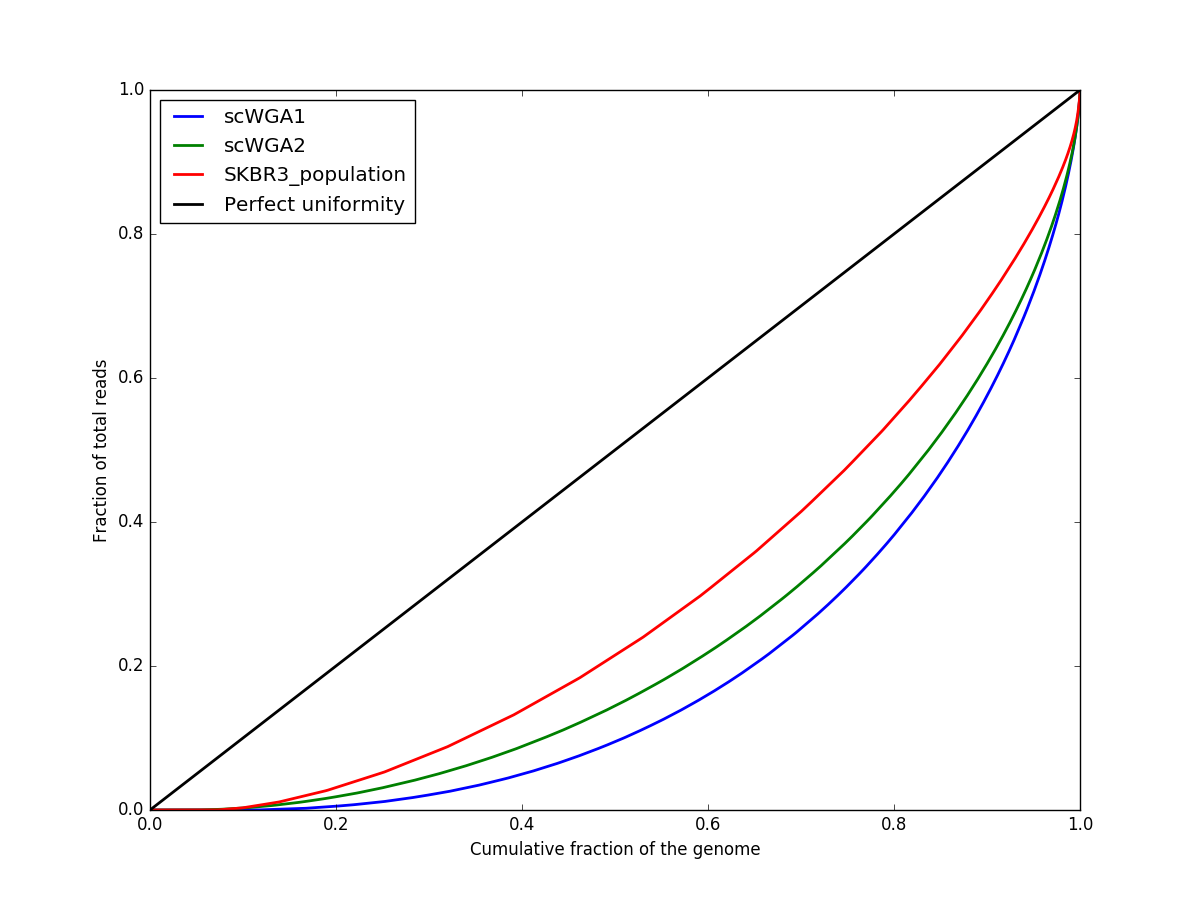
**

**Supplementary Figure S3**. Lorenz curves of coverage uniformity of SAGs obtained from single SK-BR-3 nuclei, showing the relationships between the cumulative fractions of the genome covered (x-axis) and the cumulative fraction of mapped reads (y-axis). Two SAG data (scWGA (sample1) and scWGA2 (sample2)) were compared with Bulk cell population data reported by Wang, Y. *et al.* (*Nature* **512**, 155-160, (2014)).

**Supplementary Table S1.** Mapping statistics of sd-MDA SAGs of *E. coli* and *B. subtilis* cells

| Sample Name | 16S rRNA sequence | #Raw  reads | Mapping to  *E. coli* (%) | Mapping to  *B. subtilis* (%) | Coverage of  *E. coli* genome | Coverage of  *B. subtilis* genome | Percent chimera  on *E. coli* (%) | Percent chimera  on *B. subtilis* (%) |
| --- | --- | --- | --- | --- | --- | --- | --- | --- |
| e1 | *E. coli* | 1261966 | 94.32 | 1.27 | 63.73 |  | 7.12 |  |
| e2 | *E. coli* | 1162922 | 98.55 | 1.17 | 81.00 |  | 7.11 |  |
| e3** | *E. coli* | 1144146 | 92.75 | 1.23 | 40.94 |  | 7.24 |  |
| e4 | *E. coli* | 1067261 | 92.55 | 0.92 | 67.60 |  | 5.95 |  |
| e5 | *E. coli* | 1310424 | 93.59 | 4.19 | 80.61 |  | 6.60 |  |
| e6 | *E. coli* | 769260 | 97.70 | 1.23 | 69.76 |  | 7.66 |  |
| e7** | *E. coli* | 1456037 | 97.06 | 1.13 | 44.38 |  | 6.89 |  |
| e8** | *E. coli* | 336895 | 98.90 | 4.09 | 49.23 |  | 7.20 |  |
| eA | *E. coli* | 992218 | 97.53 | 1.65 | 85.53 |  | 8.85 |  |
| eB | *E. coli* | 1046393 | 97.03 | 3.01 | 78.98 |  | 9.90 |  |
| eC | *E. coli* | 1038935 | 98.43 | 1.03 | 84.13 |  | 9.33 |  |
| eD | *E. coli* | 1187796 | 97.10 | 3.88 | 73.39 |  | 9.22 |  |
| eE | *E. coli* | 961139 | 99.21 | 1.18 | 85.05 |  | 9.96 |  |
| eF | *E. coli* | 1263083 | 99.13 | 2.10 | 80.45 |  | 9.88 |  |
| eG | *E. coli* | 1337272 | 94.74 | 2.80 | 75.86 |  | 9.55 |  |
| eH | *E. coli* | 1164487 | 95.64 | 2.10 | 65.40 |  | 10.87 |  |
| b1 | *B. subtilis* | 1154021 | 1.84 | 98.64 |  | 90.88 |  | 8.35 |
| b2 | *B. subtilis* | 991791 | 2.03 | 99.75 |  | 99.12 |  | 8.14 |
| b3 | *B. subtilis* | 994128 | 2.62 | 99.54 |  | 83.66 |  | 7.06 |
| b4** | *B. subtilis* | 836875 | 1.93 | 99.62 |  | 41.39 |  | 8.20 |
| b5 | *B. subtilis* | 1176520 | 1.89 | 99.78 |  | 91.69 |  | 9.16 |
| b6 | *B. subtilis* | 1154540 | 1.99 | 99.82 |  | 88.14 |  | 9.32 |
| b7 | *B. subtilis* | 1247368 | 2.04 | 99.79 |  | 88.02 |  | 9.03 |
| b8 | *B. subtilis* | 1224773 | 1.91 | 99.73 |  | 92.06 |  | 9.43 |
| b9 | *B. subtilis* | 1032817 | 1.80 | 99.77 |  | 75.97 |  | 7.59 |
| b10** | *B. subtilis* | 1081900 | 0.77 | 99.79 |  | 15.53 |  | 6.49 |
| b11 | *B. subtilis* | 1063847 | 2.15 | 99.80 |  | 90.48 |  | 7.12 |
| b12 | *B. subtilis* | 1157936 | 2.03 | 99.84 |  | 80.45 |  | 8.09 |
| bA | *B. subtilis* | 1061905 | 1.84 | 99.78 |  | 89.96 |  | 7.03 |
| bB | *B. subtilis* | 1090950 | 1.49 | 99.75 |  | 88.28 |  | 7.26 |
| bC | *B. subtilis* | 993230 | 1.96 | 99.75 |  | 90.88 |  | 7.09 |
| bD | *B. subtilis* | 1016159 | 1.91 | 99.78 |  | 88.33 |  | 7.81 |
| *E. coli* gDNA* | *E. coli* | 2118576 | 93.75 | 1.94 | 99.80 |  | 0.16 |  |
| In-tube MDA1* | *E. coli* | 1196450 | 63.74 | 2.10 | 90.56 |  | 10.65 |  |
| In-tube MDA2* | *E. coli* | 1079884 | 49.72 | 1.87 | 72.61 |  | 9.57 |  |
| In-tube MDA3* | *E. coli* | 1549742 | 48.42 | 1.41 | 73.48 |  | 11.86 |  |
| cd-MDA1* | *E. coli* | 1367906 | 91.28 | 0.59 | 94.84 |  | 1.98 |  |
| cd-MDA2* | *E. coli* | 1438867 | 91.83 | 0.54 | 96.83 |  | 1.85 |  |
| cd-MDA3* | *E. coli* | 1653609 | 88.71 | 0.24 | 97.66 |  | 1.56 |  |

*Nishikawa et al. (*PLoS One* **10**, e0138733, (2015)), ** Outliners expected in Figure 3d

**Supplementary Table S2.** *De novo* assembly of sd-MDA SAGs of *E. coli*

|  | sd-MDA | cd-MDA | In-tube MDA |
| --- | --- | --- | --- |
| # Contig (≥500 bp) | 835 ± 127 | 1194 ± 160 | 4082 ± 280 |
| Total length (kbp) | 3064 ± 580 | 4903 ± 81 | 7584 ± 441 |
| N50 | 13000 ± 1993 | 13205 ± 2982 | 3833 ± 137 |
| Statistics with reference genome |  |  |  |
| Genome recovery (%) | 63 ± 12 | 90 ± 1 | 66 ± 9 |
| Fully unaligned contig (%) | 4.23 ± 1.29 | 39.8 ± 1.95 | 72.2 ± 3.05 |
| Contamination (%) | 0.42 ± 0.28 | 3.2 ± 0.55 | 28 ± 4.4 |

Genome recovery rates were determined by Bowtie using contigs ≥ 500bp. Contamination rates were determined by CheckM using contigs ≥ 1000bp. The other parameters were determined by QUAST.

**Supplementary Table S3.** Whole genome sequencing analysis of sd-MDA SAGs from single SK-BR-3 cells

|  | Sample 1 | Sample 2 |
| --- | --- | --- |
| Sequence read mapped to reference (%) | 99.6 | 99.5 |
| Mean map depth (X) | 21.0 | 24.0 |
| Human genome coverage (%) | 88.8 | 95.1 |
| Allelic dropout rate (ADO) (%) | 28.7 | 19.2 |
| False positive rate (FPR) | 5.2 × 10^-3^ | 4.6 × 10^-3^ |

**Supplementary Table S4.** Features of 17 Soil Single-cell assemblies obtained from sd-MDA

| Sample ID | Readcount | # Contig  (≥500 bp) | Total contig length (bp) | Largest contig (bp) | N50 | GC% | Phylum (AMPHORA2) | Amphora hitting assigned taxa | CheckM completeness （Phylum） | CheckM contamination (Phylum) | Soil metagenome OTU (Phylum) identified from contigs | Identity (%) |
| --- | --- | --- | --- | --- | --- | --- | --- | --- | --- | --- | --- | --- |
| Soil_1_1 | 1394877 | 848 | 4576484 | 94980 | 23215 | 50.25 | Bacteroidetes | 24 of 24 | 74.06 | 1.6 | Bacteroidetes (OTU122) | 100 |
| Soil_1_2 | 1359791 | 567 | 1898950 | 39000 | 11458 | 39.94 | Proteobacteria | 28 of 28 | 81.36 | 4.2 | Proteobacteria (OTU2857) | 95 |
| Soil_1_3 | 829886 | 475 | 1966413 | 81503 | 17773 | 48.36 | Bacteroidetes | 22 of 22 | 39.02 | 0.85 | Firmicutes (OTU1463) | 98 |
| Soil_2_1 | 904432 | 268 | 1395528 | 49699 | 12097 | 40.83 | Proteobacteria | 24 of 24 | 68.07 | 5.12 | Proteobacteria (OTU1493) | 92 |
| Soil_2_2 | 794264 | 250 | 1060900 | 60976 | 11686 | 44.77 | Chlamydiae | 17 of 17 | 62.05 | 3.79 | Chlamydiae (OTU1633) | 94 |
| Soil_2_3 | 691465 | 482 | 1197713 | 33678 | 8238 | 55.34 | Planctomycetes | 3 of 4 | 20.35 | 0 | - | - |
| Soil_2_6 | 1615733 | 359 | 562000 | 37227 | 3454 | 35.77 | Proteobacteria | 3 of 7 | 16.6 | 2.94 | - | - |
| Soil_2_7 | 1771979 | 494 | 1337160 | 20716 | 5734 | 35.53 | Acidobacteria | 1 of 4 | 6.97 | 0.36 | Candidatus Saccharibacteria (OTU712) | 100 |
| Soil_2_11 | 1710248 | 1085 | 3395009 | 76684 | 13501 | 46.99 | Proteobacteria | 16 of 29 | 86.55 | 53.9 | Cyanobacteria (OTU4393) | 92 |
| Soil_2_12 | 1126930 | 148 | 760861 | 46732 | 15617 | 45.86 | Proteobacteria | 7 of 27 | 59.76 | 2.42 | Parcubacteria (OTU2253) | 99 |
| Soil_2_13 | 1879416 | 1022 | 2234083 | 45847 | 5511 | 46.29 | Proteobacteria | 15 of 27 | 59.31 | 20.7 | Cyanobacteria (OTU4393) | 92 |
| Soil_2_14 | 1266723 | 419 | 1937495 | 54957 | 13243 | 39.93 | Proteobacteria | 6 of 8 | 58.95 | 0 | Proteobacteria (OTU46) | 100 |
| Soil_2_15 | 1282480 | 104 | 695754 | 60907 | 28522 | 34.68 | Firmicutes | 9 of 27 | 50.85 | 2.92 | Firmicutes (OTU2828) | 74 |
| Soil_2_24 | 1798897 | 182 | 361776 | 32546 | 8405 | 38.82 | Proteobacteria | 2 of 2 | 14.5 | 0.84 | - | - |
| Soil_2_26 | 1097946 | 1768 | 2072888 | 21604 | 1275 | 40.86 | Proteobacteria | 14 of26 | 60.52 | 32.51 | Firmicutes (OTU1195) | 91 |
| Soil_2_28 | 1183943 | 405 | 1126304 | 35624 | 8108 | 39.97 | Verrucomicrobia | 23 of 26 | 54.79 | 3.41 | Verrucomicrobia (OTU1837) | 100 |
| Soil_2_30 | 1457036 | 427 | 1681710 | 27974 | 7776 | 41.36 | Chlamydiae | 30 of 30 | 83.82 | 3.01 | Chlamydiae (OTU38) | 100 |

**Supplementary Table S5.** Comparison of platforms for single-cell whole genome amplification (WGA) toward single-cell sequencing

| Platform Name | Reaction format | Reaction scale | Number of sample/run | Time | Throughput | Pros | Cons |
| --- | --- | --- | --- | --- | --- | --- | --- |
| Conventional FACS-based MDA (1 and 2) | Multi-well plate (96 to 384 well) | 15 µL | ~384 cell | 1 day | 16 cell/h | - Tool for use with both mammalian cell and bacteria - Combination with liquid dispensing system and multi-well plate | - Low throughput reaction - Large reaction volume - Low amplification success rate |
| Fluidigm C1 WGA (3) | Microfluidic channel circuit | 270 nL | ~96 cells | 9.5 h | 10 cells/h | - Automated regent mixing and thermal reaction in microfluidic channel - Small reaction volume - Unbiased and contamination-less amplification | - Tool for use with mammalian cell only (5-25 μm) - Low throughput reaction |
| MIDAS (4) | Microfabricated microwell | 12 nL | 408 cells | 16 h | 25.5 cells/h | - Tool for use with both mammalian cell and bacteria - Small reaction volume - Unbiased and contamination-less amplification - High coverage breadth | - Low throughput WGA reaction - Manual reagent injection into microwells - Requirement of 2nd round amplification for subsequent analysis |
| in-gel digital MDA (5) | In-gel matrix | 60 nL | 206 cells | 12 h | 17 cells/h | - Simple reaction handling - Small reaction volume - Chimera-less amplification - High coverage breadth | - Tool for use with bacteria only - Low throughput reaction - Requirement of 2nd round amplification for subsequent analysis |
| cd-MDA (6) | Microfluidic droplet | 67 pL  (total 10 µL) | 1 cell | 3 h | 0.3 cells/h | - Ultra-small reaction volume - Unbiased and contamination-less amplification - High coverage breadth | - Tool for use with bacteria only - Low throughput reaction - Requirement for microfluidic operation |
| sd-MDA  (this study) | Microfluidic droplet | 240 pL | 84,000 cells | 4 h | 21,000 cells/h | - Tool for use with both mammalian cell and bacteria - Ultra-small reaction volume - High throughput and semi-automated reaction by microfluidic droplet fusion - Contamination-less amplification - High coverage breadth | - Requirement of 2nd round amplification for subsequent analysis - Requirement for microfluidic operation |

(1) Rinke, C. *et al. Nat Protoc* **9**, (2014), (2) Wang, Y. et al. *Nature* ***512***, (2014), (3) de Bourcy, C. F. *et al.* *PLoS One* **9**, (2014), (4) Gole, J. *et al.* *Nat Biotechnol* **31**, (2013), and (5) Xu, L., *et al.* *Nat Methods* **13**, (2016), and (6) Nishikawa, Y., *et al.* *PLoS One* **10**, (2015).
